# Crazing Initiation and Growth in Polymethyl Methacrylate under Effects of Alcohol and Stress

**DOI:** 10.3390/polym15061375

**Published:** 2023-03-09

**Authors:** Ying Yan, Yujia Sun, Jiyang Su, Bo Li, Ping Zhou

**Affiliations:** State Key Laboratory of High-Performance Precision Manufacturing, Department of Mechanical Engineering, Dalian University of Technology, Dalian 116024, China

**Keywords:** crazing, swelling, tensile test, plastic deformation

## Abstract

Polymer crazing is typically a precursor to damage and considerably reduces the mechanical performance of polymer materials. The concentrated stress caused by machines and the solvent atmosphere created during machining exacerbates the formation of crazing. In this study, the tensile test method was employed to examine the initiation and progression of crazing. The research focused on polymethyl methacrylate (PMMA), both regular and oriented, and the impact of machining and alcohol solvents on the formation of crazing. The results showed that the alcohol solvent influenced PMMA through physical diffusion, whereas machining primarily affected crazing growth via residual stress. Treatment reduced the crazing stress threshold of PMMA from 20% to 35% and produced a threefold increase in its sensitivity to stress. The findings revealed that oriented PMMA exhibited 20 MPa higher resistance to crazing stress compared with regular PMMA. The results also indicated that the extension of the crazing tip and thickening were in conflict, with the crazing tip of regular PMMA severely bending under tensile stress. This study provides valuable insight into the initiation of crazing and the methods of its prevention.

## 1. Introduction

Polymer materials, including polymethyl methacrylate (PMMA), are widely used due to their favorable material properties. PMMA, in particular, is favored in the production of optical lenses and fighter cockpit covers as it is lighter, more resistant to corrosion, and boasts better optical and mechanical properties than quartz glass [1,2,3,4,5,6]. Crazing, however, is a ubiquitous mechanism of plastic deformation that occurs in brittle polymeric materials, including PMMA, and is caused by local plastic deformation under tensile stress. This form of damage, characterized by a tensile-stress-induced subscale crack, negatively impacts PMMA’s optical properties, reducing light transmittance, affecting contrast, and causing optical distortion, which eventually leads to mechanical fracture [7]. Traditional shaping methods are not capable of producing PMMA products with the high surface accuracy and low surface roughness that is required for complex free-form surfaces, necessitating mechanical processing. However, the high level of local stress concentration during the machining and solvent cleaning processes exacerbates the formation of crazing, reducing the stress threshold for crazing initiation and triggering its development [8,9].

Exposure to a solvent atmosphere over extended periods of time leads to a reduction in the threshold for crazing and an increase in the density of crazing in PMMA. Roger P. Kambour and E.J. Kramer proposed that crazing at the crack tip in PMMA could undergo as much as 100% elastic deformation under stress before the crack propagation resumed. This deformation is estimated to account for as much as 40% of the nominal Griffith energy of crack propagation. The thickening process of methanol-induced crazing, which can result in thickness increases from 40% to 140%, absorbs a substantial amount of energy and can lead to material damage [10,11]. Numerous studies have been conducted to evaluate the impact of the solvent atmosphere on PMMA. For instance, E. H. Andrews et al. employed a creep experiment to investigate the effect of different alcohol solvents on PMMA and found that physical diffusion, rather than chemical reactions, occurred due to the large molecular weight difference between PMMA and the alcohol solvents [12]. This caused local cavities to form in the PMMA molecular chains, reducing its mechanical properties and crazing stress resistance. In another study, Kramer, Edward J. measured the kinetics of craze growth in PMMA under contact with liquid methanol and found that solvent crazing velocity was limited by the hydrodynamic transport of the solvent through a porous craze structure [13]. N. L. Thomas and A. H. Windle also studied the characteristics of methanol diffusion in PMMA and found that a concentration gradient was generated, leading to internal local stress that exceeded the stress required for crazing to occur [14]. Luo et al. investigated the diffusion between PMMA and solvent molecules and discovered that diffusion caused volume expansion and surface distortion in PMMA, leading to stress concentration [15]. The crack expansion rate of polyethylene under different environmental conditions was studied by Maximilian Thuy et al., who showed that environmental factors played a role in the crack expansion rate [16]. The effects of natural weathering on the mechanical properties of PMMA, ABS, and ASA were studied by Sánchez-Calderón, I et al., who reported that the natural environment and light considerably reduced the mechanical properties of PMMA [17]. The diffusion effect of solvents such as sedimentary oil and TPP on the crazing growth of PMMA was studied by Masoud Razavi et al., who found that the diffusion of these solvents promoted molecular chain aggregation and the nucleation of PMMA, leading to voids in the molecular network and high rates of crazing, cracking, and brittle fracture [18]. Despite these studies, research is lacking on the changes experienced by PMMA after diffusion and the growth of crazing, a suitable theoretical model describing the effect of the solvent atmosphere on the crazing process is also lacking.

The machining process induces concentrated stress, affecting the initiation and development of crazing. H. Michler studied the size, shape, and internal structure of crazing in PMMA, PS, PC, and other machined materials before and after annealing [19]. Non-annealed PMMA, when subjected to an external force quickly, nucleated, forming micro-fibrous and microcavity structures. PMMA exhibited strong strain softening under tensile stress, indicating crazing. Gao et al. monitored the progression of crazing damage using acoustic emission and finite element simulation and found that growth was uneven and nonlinear, and the constitutive models of crazing and microcracking were obtained [20]. Razavi et al. and Wang Y et al. also investigated the impact of mechanical forces on the growth of crazing and cracking in PS, PMMA, and other materials. Machining was found to improve PMMA creep deformation, resulting in irreversible deformation and the expansion of cracks with associated crazing [18,21]. The surface damage caused by machining can promote the crazing formation process by accelerating the development and coalescence of micro-voids, which can ultimately lead to material fracture [22]. This limitation substantially affects the development of polymer material machining, as processing defects and vibrations induced by machining can further contribute to the destruction of polymer materials during their use [23]. 

Theoretical studies on the initiation of crazing are limited. Lang RW et al. proposed a criterion for crazing initiation based on elastic strain. Kinoch AJ et al. and Jie et al. proposed criteria based on the maximum principal stress and equivalent shear stress, respectively, as per the nucleation theory [24,25,26]. S. Socrate et al. used finite element simulation and obtained the critical stress of polystyrene (PS) for the maximum principal stress criterion [27]. Wu et al. then used molecular dynamics simulations to evaluate the initiation of crazing in a PS material using this critical stress [28].

In this study, the dynamic evolution of crazing during stretching experiments on regular and oriented PMMA was analyzed. The regular and oriented PMMA samples used in the experiment underwent various treatments, including machining and exposure to solvent environments. The mechanisms of crazing initiation, growth, thickness increase, and crack development under tensile stress were also explored. To comprehend the microscopic process of crazing growth in PMMA, the crazing characteristics of both oriented and regular PMMA were compared, and the impact of the PMMA crazing state was investigated. 

## 2. Materials and Experiments

### 2.1. Materials

In this study, regular and oriented PMMA were tested using the tensile test method [29]. The experiment used alcohol (Shunxing Chemical Company, Shenyang, China), regular PMMA, and oriented PMMA as the sample materials. Regular PMMA was created through injection processing using PMMA particles with a molecular weight of about two million (PMMAIRL409 002 IRL Series, Mitsubishi Group, Tokyo, Japan). Oriented PMMA was created by heating regular PMMA materials above the glass transition temperature (Tg), stretching a plane in a specific direction, and cooling to obtain anisotropic physical and mechanical properties. As shown in Equation (1) [30], the drawn ratio was used to describe oriented PMMA.

(1) $$\lambda =\sqrt{\frac{h_{2}}{h_{1}}}$$

Here, $h_{1}$ and $h_{2}$ are the height of the vertical stretching plane before and after oriented stretching, respectively. The type of oriented PMMA used in this experiment was YB-DM-10, which has a tensile ratio of 68%. The specific acquisition process is detailed in the literature [30]. The partial mechanical properties of regular and oriented PMMA are listed in Table 1. 

### 2.2. Experiments

**Figure 1:**
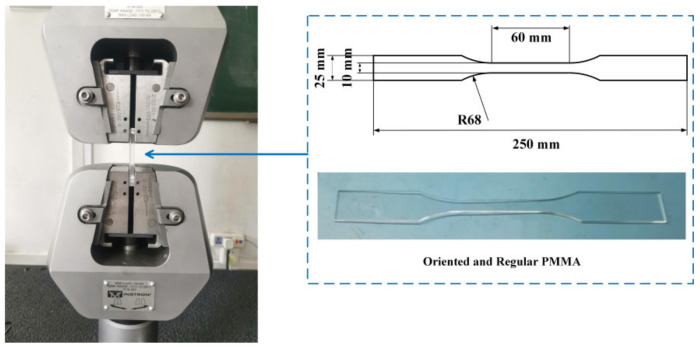
Experimental instruments and the size of regular and oriented PMMA specimens.

The size of regular and oriented PMMA specimens is depicted in Figure 1. Subject to a variety of treatments, including machining, solvent environment, and loading conditions, twelve groups of experiments were conducted with five samples per group based on the Taguchi method, as detailed in Table 2 [31]. The specimens were machined using JDHGT600T 3-Axis High-Speed Machining Centers(JDHTG Series, Beijing Jing Diao, Beijing, China) with a spindle speed of 6000 r/min and feed rate of 60 mm/min, followed by polishing to ensure better surface quality. The samples were then fully immersed in an alcohol solution and sealed for 20 days to facilitate the complete diffusion between the alcohol and PMMA. Groups 3, 7, and 10 were sealed in the air for 20 days. After 20 days of exposure to either an alcohol solution or air, uniaxial tensile stress was applied to PMMA in groups 3–4 and 7–12 using an electronic universal material testing machine (Instron 5982, Instron, Boston, MA, USA). The crazing growth state was recorded under varying loads using a polarizing microscope, whereas the stretched surface state was analyzed using a confocal microscope (VK-X250, Keyence, Osaka, Japan) and a scanning electron microscope (SU5000, HITACHI, Tokyo, Japan).

## 3. Results and Discussion

### 3.1. Effect of Solvent Atmosphere on Crazing Growth

#### 3.1.1. Effect of Solvent Atmosphere on Crazing Initiation without Tensile Stress

**Figure 2:**
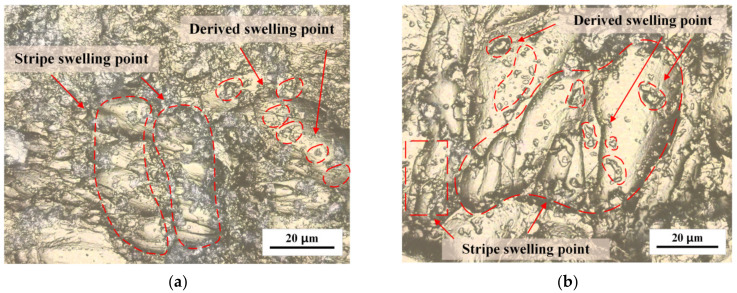
Swelling of PMMA surface without machining for (**a**) Regular PMMA; (**b**) Oriented PMMA.

In this study, the influence of the solvent environment on crazing initiation in the absence of tensile stress was evaluated in Groups 1–2 and 5–6. We observed no crazing on the surface of PMMA without external stress; however, a complex swelling phenomenon related to localized stress was observed. The experiments showed that nonmilled regular PMMA and oriented PMMA in Groups 1–2 were immersed in alcohol and showed no crazing on the surface; instead, a swelling phenomenon appeared on both regular and oriented PMMA, which was evenly distributed in strips. Figure 2a shows that the swelling points on the surface of regular PMMA were dense and unevenly distributed, with numerous smaller-scale swelling points on the surface of larger-scale swelling points and a higher density for the derived swelling points. In contrast, Figure 2b shows that oriented PMMA primarily exhibited strip swelling points that were 60–80 μm in length with a small portion of 1–3 μm which spotted the swelling points. The arrangement of swelling points in oriented PMMA was regular and generally parallel, resulting in a more directional and consistent swelling phenomenon.

**Figure 3:**
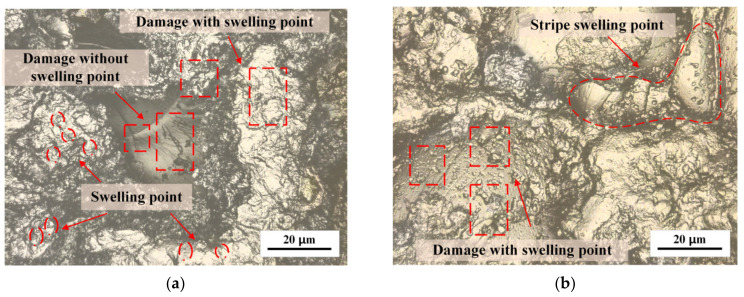
Swelling of PMMA surface after machining with (**a**) Regular PMMA (**b**) Oriented PMMA.

As depicted in Figure 3, the surface morphology of PMMA exhibited no crazing, but an increase in swelling points could be observed after machining. Machining caused concentrated stress points on the PMMA surface, influencing the crazing and swelling processes. The swelling points on PMMA in Groups 5–6, compared with the strip swelling points in Groups 1–2, were disordered in distribution. The height of the swelling points on the machined PMMA surface was elevated, and the swelling points were larger. The surface of the regular PMMA swelling points was rough and characterized by a complex morphology. Smaller derived swelling points, less than 1 μm in size, were observed to be intertwined with the machined surface and distributed around the central point. At the central point, crazing occurred in the same direction. 

In the regions of low molecular chain density in PMMA, micro-voids existed between the chains, and swelling mainly occurred in these micro voids. When PMMA came into contact with alcohol molecules, the two substances interacted through diffusion. Lisaka K proposed that the molecular weight directly affected the diffusion rate during this process [32]. Because a single PMMA molecule has a molecular weight thousands of times higher than that of an alcohol molecule, the diffusion rate of PMMA molecular chains was relatively static compared with that of alcohol molecules. Gradually, alcohol molecules were diffused into the micro-voids of PMMA, increasing the activity and kinetic energy of the PMMA molecular chains. This promoted their movement and the expansion of the region near the micro-voids, leading to the formation of swelling points. Brown H R and Kramer E J found that the internal tensile stress was the key factor leading to craze initiation by observing a mixed system of polymer and methanol [33]. The swelling points had poor surface morphology and caused concentrations and stress gradients within the matrix material surrounding them. Kausch, H.H. Dettenmaier discussed the molecular orientation at the initial stage of crazing and found that a regular molecular chain arrangement helped to establish the energy balance and reduced the possibility of the orientation mechanism transforming into crazed fiber [34]. PMMA with a more uniform molecular chain distribution, such as oriented PMMA, would be less susceptible to the formation of micro-voids during interactions with alcohol, reducing the likelihood of crazing and the associated crazing stress.

#### 3.1.2. Effect of Solvent Atmosphere on Crazing Initiation with Tensile Stress

A comparison was made between the initiation and progression of crazing in Groups 3–4 under different solvent atmospheres, with and without 95% alcohol by volume. The term “stress at crazing” was used to refer to the stress level at which crazing began to occur, and the “sensitivity of crazing to stress” was defined as the ratio of the increase in crazing length to the increase in applied stress. The paper notes that all tensile stress was applied vertically.

**Figure 4:**
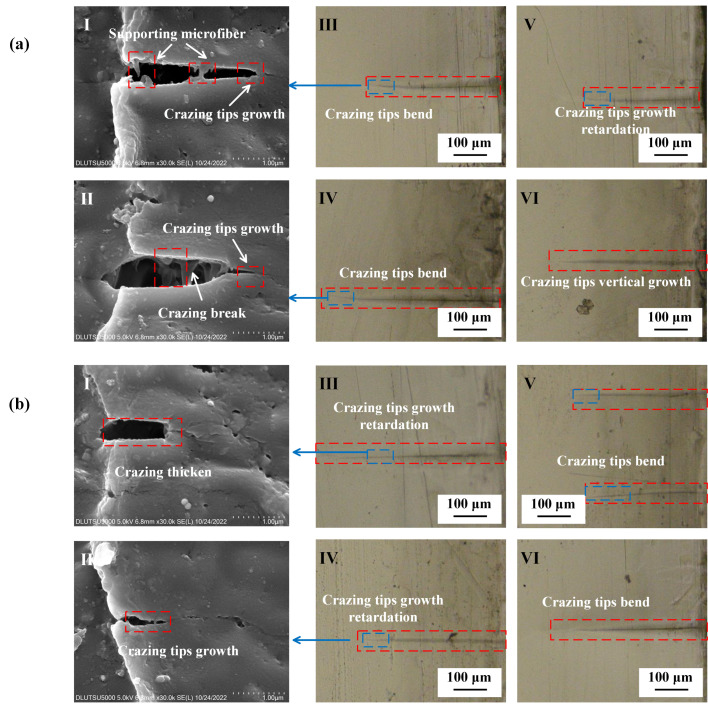
PMMA crazing growth state under tensile stress only. (**a**) Oriented PMMA. **I**: Crazing tip growth and supporting microfiber; **II**: crazing tip growth and break; **III**–**V**: crazing tip bend; **VI**: crazing tip growth retardation. (**b**) Regular PMMA. **I**: Crazing thickening and not supporting microfiber; **II**: crazing tip growth and slowly thickening; **III**,**IV**: crazing tip growth retardation; **V**,**VI**: crazing tips circuitously growth.

Figure 4 presents the observation of crazing under tensile stress conditions. The results revealed that the crazing in oriented PMMA occurred perpendicular to the direction of tensile stress with a size ranging from 300 to 400 μm. The forward expansion of the crazing tip was primarily responsible for the growth in crazing, resulting in a slight increase in thickness. The width of the crazing was approximately 1 μm, and some derived crazing was observed. Regular PMMA also showed crazing perpendicular to the direction of tensile stress with a size ranging from 200 to 300 μm, but there was no derived crazing observed around the crazing. The formation of crazing and derived crazing on the surface of PMMA is linked to the entanglement network formed by the molecular chain. Kramer E J proposed a positive correlation between chain contour length *le* between entanglements and the extension ratio *λ* [35]. After directional stretching, the *le* of the oriented PMMA was much larger than that of regular PMMA, which also accounted for the longer crazing growth length in the former. Furthermore, Kramer E J and Donald A M jointly proposed that materials with a regular molecular chain arrangement were more likely to experience a large-scale entanglement motion during crazing formation [36]. As crazing was more easily generated with plane strain than plane stress, more derived crazing occurred around the oriented PMMA, which was primarily caused by the plastic deformation of the main crazing. The SEM results indicated that the crazing tip in oriented PMMA had a strong tendency to extend forward, and this extension resulted in the breaking of the supporting microfibers and the thickening of crazing along with the formation of derived crazing. Additionally, the crazing tip in regular PMMA did not show any forward extension, and the breaking of the supporting micro-fibers caused the crazing to be relatively regular.

**Figure 5:**
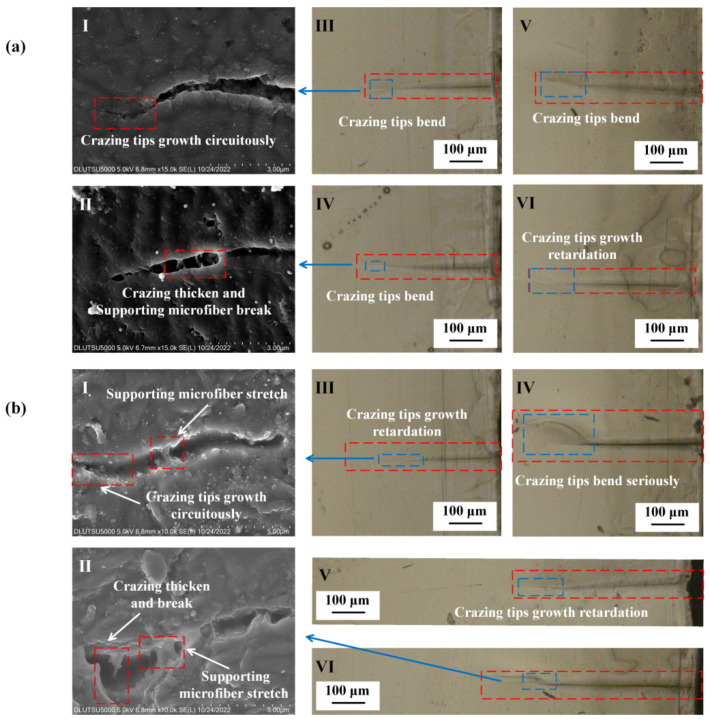
PMMA crazing growth state under tensile stress after alcohol treatment. (**a**) Oriented PMMA. **I**: Crazing tip circuitously grow; **II**: crazing slowly thickens and supports microfiber breaks; **III**–**V**: crazing tips bend; **Ⅵ**: growth retardation of crazing tips. (**b**) Regular PMMA. **I**: crazing tips circuitously grow, and supporting microfiber stretches; **II**: supporting microfiber stretches and breaks; **III**: growth retardation of crazing tips; **IV**: crazing tips serious bend. **V**: growth retardation of crazing tips; **VI**: crazing tips bend, ultimately perpendicular to the direction of tensile stress.

The results of the alcohol treatment on PMMA under tensile stress are depicted in Figure 5. The crazing tips of both oriented and regular PMMA were observed to bend after alcohol exposure, causing a deviation from their perpendicular alignment with the direction of applied stress. There was a noticeable increase in the thickness of the crazing, with the end of the crazing on regular PMMA breaking, and this resulted in the formation of micro-cracks. The size of crazing in the oriented PMMA was measured between 300 and 400 μm, whereas the crazing on regular PMMA was characterized by a staggering pattern, with lengths of 400–500 μm and widths of 5–10 μm. The orientation of the crazing in the oriented PMMA changed only in size and growth direction. As the crazing tip continued to extend, thickening and fracture occurred. The internal micro-fibrils within the crazing were still visible. Furthermore, the center of the crazing in regular PMMA was found to be much larger than that of oriented PMMA, with a thicker and stronger tendency to give rise to additional crazing.

**Figure 6:**
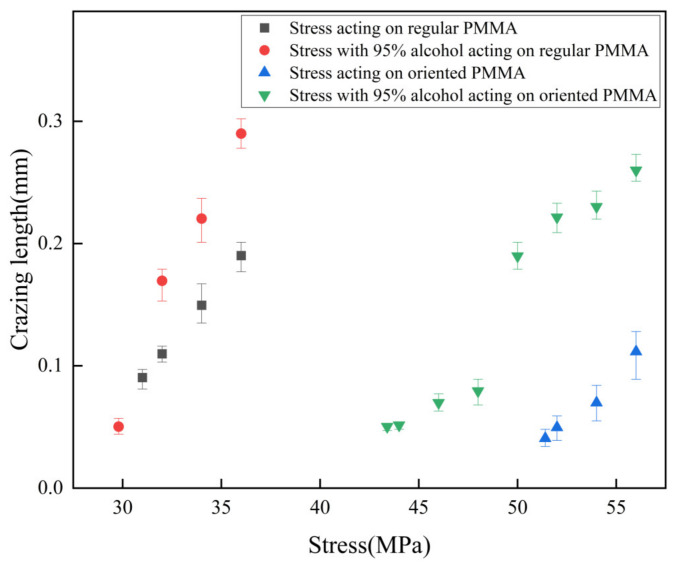
Effect of alcohol on crazing growth of PMMA without machining.

Figure 6 presents the relationship between the crazing length and stress, both before and after alcohol treatment, in regular and oriented PMMA obtained using a polarizing microscope. The results are summarized in Table 3, which displays the stress at which crazing occurred alongside the sensitivity of the crazing to stress. The stress value required for crazing to occur in regular PMMA decreased from 31.4 to 29.8 MPa after 95% alcohol treatment, representing a decrease of 1.6 MPa, which is an insignificant change. In contrast, the stress value in oriented PMMA fell from 51.4 to 43.4 MPa, highlighting a decrease of 8.0 MPa. This demonstrates that oriented PMMA exhibited superior resistance to crazing stress and reduced sensitivity to alcohol compared with regular PMMA. The growth of the crazing was observed to be stable.

The sensitivity of crazing to stress in regular PMMA increased from 0.049 mm/MPa after 95% alcohol treatment, and a linear relationship between the crazing length and tensile stress could still be observed. By contrast, the sensitivity of crazing to stress on the surface of oriented PMMA increased from 0.015 to 0.055 mm/MPa after alcohol treatment, and the relationship between the crazing length and tensile stress was no longer linear. A localized increase in sensitivity was observed when crazing occurred within a narrow range of stress (48.5–50.3 MPa), with a sensitivity of 0.071 mm/MPa, which was higher than that observed under other stress states (0.023 mm/MPa).

Following alcohol treatment, the local expansion of the PMMA surface resulted in local plastic deformation and concentrated stress, lowering the material’s anti-crazing stress. The effect of concentrated stress caused the crazing to rapidly grow when it passed through the swelling points during the growth process, indicating a high sensitivity to stress. In the regular PMMA, the distribution of the molecular chains was nonuniform, and there were microcavities between the molecular chains. Crazing was caused by the combination of microcavities and a break in the molecular chain. The concentrated stress caused by alcohol treatment resulted in a complex stress gradient with different stress gradient directions and sizes. During the growth process, the tip of the crazing bent was due to the combined effect of tensile and concentrated stresses. Additionally, the concentrated stress could also promote the generation of derived crazing. After directional stretching, the molecular chain distribution of the oriented PMMA became more uniform. The result of the concentrated stress was stress gradients that were close to each other in all directions. After alcohol treatment, the crazing tip of the oriented PMMA did not bend as much as that of regular PMMA. The crazing tip still had a strong tendency to extend forward. However, crazing would continue to rapidly grow rapidly when passing through the concentrated stress points, demonstrating the crazing’s high sensitivity to tensile stress. 

### 3.2. Effect of Concentrated Stress on Crazing Initiation

**Figure 7:**
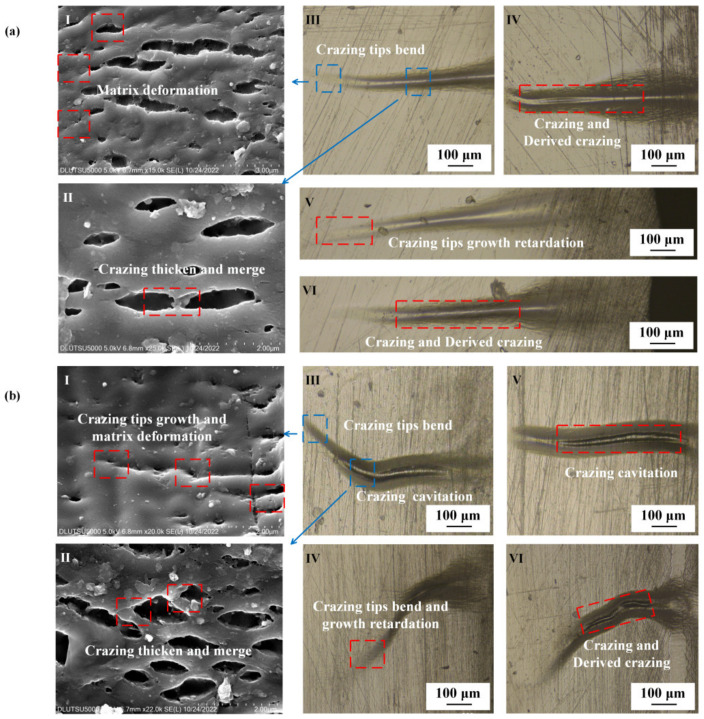
Crazing growth state of PMMA after milling under tensile stress. (**a**) Oriented PMMA. **I**: Crazing tip growth retardation and matrix deformation; **II**: crazing slowly thickening and supporting microfiber breaking; **III**,**IV**: crazing tip bending, causing derived crazing; **V**: crazing tip growth retardation. **VI**: large amount of derived crazing. (**b**) Regular PMMA. **I**: Crazing tip growth and matrix deformation; **II**: crazing thickening and merging; **III**: crazing tip bending and cavitation; **IV**: crazing tip bending and growth retardation; **V**: crazing cavitation; **VI**: resulting large amount of derived crazing.

Figure 7 depicts the effect of machining on the crazing of the PMMA surface under tensile stress. The length of the crazing increased to between 400 and 700 μm, and its thickness increased to 10–100 μm after machining. A large amount of derived crazing was produced on both sides of the main crazing, and the direction of the crazing remained perpendicular to the direction of tensile stress. The tip of the crazing continued to extend forward, but at a reduced rate, as the length increased and the thickness grew. The tip was observed as bent, and the end of the crazing broke into a broad area. The interior of the crazing comprised numerous regular microcavities of varying sizes, and the tip continued to form microcavities and merge. The crazing on the surface of regular PMMA was severely bent; the total length of the main crazing was 500–900 μm, and the thickness was larger than 100 μm. The crazing growth direction was no longer perpendicular to the tensile stress direction, but the interior of the main crazing was still composed of a large amount of small crazing that was perpendicular to the tensile stress direction. The distance from the end of the crazing to the tip of the crazing was the measured crazing length. A large amount of derived crazing appeared on both sides, and the density of crazing in the regular PMMA was substantially higher than that of the oriented PMMA. The microcavities in the interior of the crazing had lost their regularity. To form the crack, the crazing on the surface of regular PMMA broke and merged with the larger-scale cavities.

**Figure 8:**
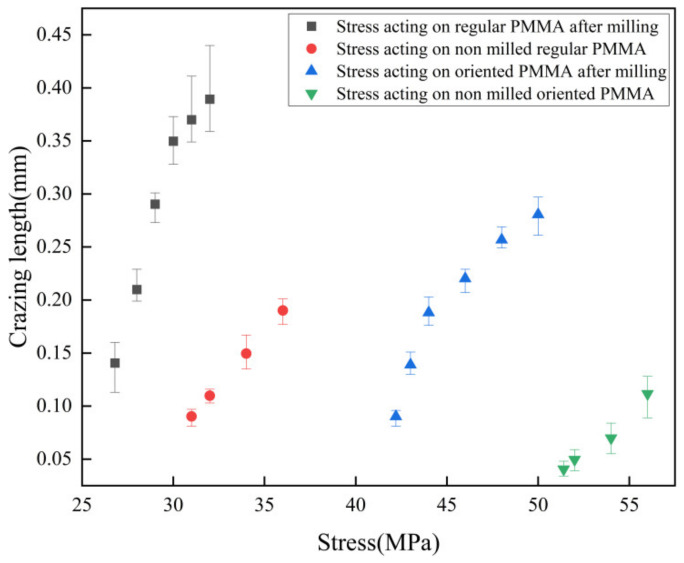
PMMA crazing growth state before and after machining.

The results of the study, shown in Figure 8 and Table 4, indicate that machining caused a decrease in the stress value at which crazing occurred in both regular and oriented PMMA. The decrease was 4.6 MPa for regular PMMA and 9.2 MPa for oriented PMMA, with the latter appearing more sensitive to machining. The sensitivity of crazing to stress in regular PMMA increased from 0.02 to 0.065 mm/MPa and, in oriented PMMA, from 0.015 to 0.04 mm/MPa. In the absence of machining, a linear relationship existed between the crazing and tensile stress, and the sensitivity was stable. However, after machining, crazing was more sensitive to stress at the initial stages of growth and experienced rapid growth as tensile stress increased. As the tensile stress continued to increase, the sensitivity of crazing to stress decreased, and its growth slowed, eventually breaking and transforming into a microcrack when reaching a length of 0.3–0.4 mm. The growth of the crazing was limited by the increasing thickness of the crazing and resulted in reduced sensitivity to stress.

For brittle PMMA materials, the coexistence and competition between shear and craze are the main damage mechanisms. On the undamaged surface, plastic deformation at the craze tip can partially inhibit the crazing process. However, surface damage induced by machining substantially reduced the stress that was required for craze initiation in materials [37]. Bucknall C. B. proposed the theoretical model of surface defects, which suggested that at the damage point, a surface defect was analogous to embedding a particle that could continuously exert stress on the surrounding material [38]. The initiation of crazing could be considered a frustrated fracture process rather than a yielding mechanism, as the presence of the surface defects created localized stress concentrations that could promote the formation and propagation of multiple crazing. This phenomenon was especially notable in brittle materials such as PMMA, where the interplay between shear plastic deformation and crazing failure was characterized by competition and coexistence, and the presence of surface damage further exacerbated this complex behavior. Residual stress was prevalent at the damage points in this experiment, and this stress state facilitated the development of frustrated fracturing under tensile loading, leading to a notable reduction in the anti-cracking stress of both oriented and regular PMMA materials. Calculations showed that a strain energy release rate (G_nasc) of less than 1 J/m was sufficient to generate a typical nascent craze with a thickness of 20 nm, which explained the low-stress requirement of only 20–30 MPa, which was able to cause crazing.

Under tensile stress, the crazing on the surface of oriented PMMA remained perpendicular to the direction of the stress, whereas regular PMMA experienced considerable bending. Saad-Gouider N proposed, through simulation, that the existence of surface defects could cause multiple crazes to form in different directions [39]. The rate and direction of multiple craze growth were affected by the size and direction of the stress gradient at the surface defect point. In this experiment, the anisotropic stress distribution at the surface defect of regular PMMA caused the bending phenomenon of crazing when the resultant force direction and the external stress direction were deflected.

### 3.3. Effects of Concentrated Stress and Solvent Atmosphere on PMMA Crazing Initiation

**Figure 9:**
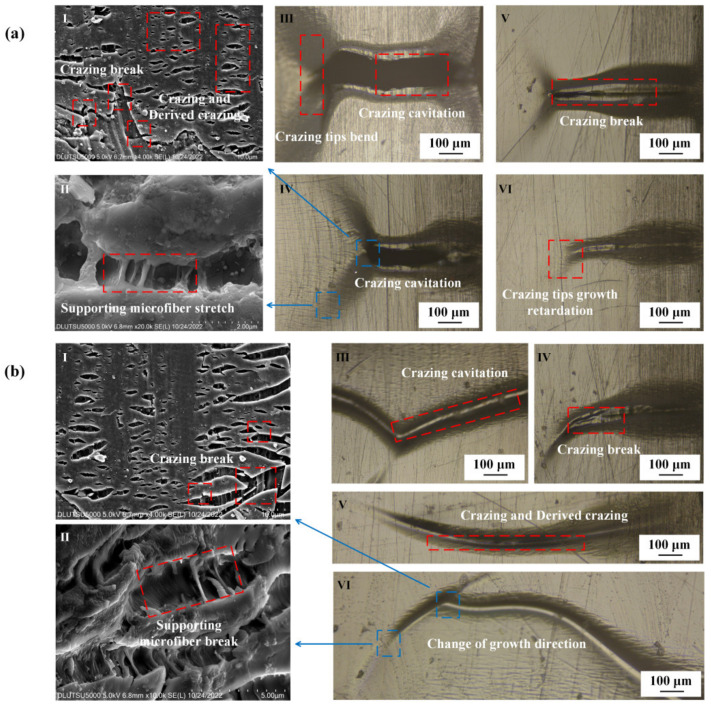
PMMA crazing state under the combined action of alcohol and machining. (**a**) Oriented PMMA. **I**: Crazing tip growth and matrix deformation; **II**: supporting microfiber stretching; **III**,**IV**: crazing cavitation; **V**,**VI**: crazing tip growth retardation. (**b**) Regular PMMA. **I**: Crazing breaking; **II**: supporting microfiber stretching and breaking; **III**: crazing cavitation, causing derived crazing; **IV**: crazing break; **V**: crazing thickening, causing derived crazing; **VI**: crazing tip growth retardation, and the growth direction suddenly changing.

Figure 9 shows the state of PMMA surface crazing growth caused by the combined action of alcohol and machining. As shown in Figure 9a, the crazing on the surface of oriented PMMA was broken, and the cavity after the break was 500 μm long and 150 μm wide. The method for determining the crazing length was the same as that shown in Figure 7. The forward extension of the crazing tip is usually bent in two different directions. At the bending crazing tip, the crazing still grew forward, and the supporting microfiber at the crazing tip was generated and broke. As shown in Figure 9b, the scale of cavities on the surface of the regular PMMA was smaller, but the direction of the forward extension of the crazing tip was more random. The crazing in the cavity’s front was also broken, but the crazing was mostly perpendicular to the tensile stress. The tip of the crazing was no longer perpendicular, and many of the supporting micro-fibers were broken.

**Figure 10:**
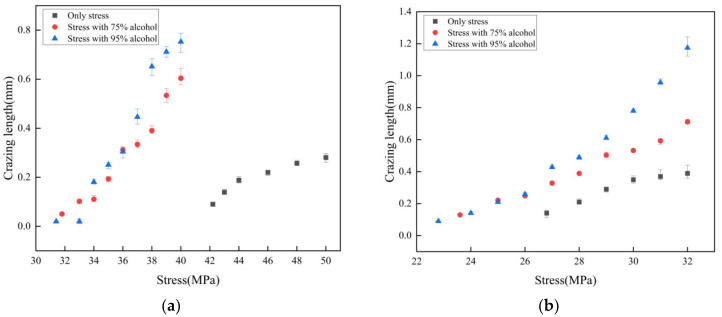
Crazing growth state under the combined action of alcohol and machining: (**a**) Oriented PMMA; (**b**) Regular PMMA.

Figure 10 illustrates the state of the crazing growth in both oriented and regular PMMA after being subjected to treatment with varying concentrations of alcohol (75% and 95% by volume) and air. The results presented in Table 5 reveal the stress levels at which crazing occurred, as well as its sensitivity to stress. Following machining, the stress required to induce crazing in oriented PMMA decreased from 42.2 to 31.8 and 31.4 MPa after being treated with 75% and 95% alcohol, respectively. A similar decrease was observed in regular PMMA, with the stress dropping from 26.8 to 23.4 and 22.8 MPa after treatment with the two alcohol concentrations, respectively. We found that treating PMMA with alcohol substantially reduced the tensile stress that was required to cause crazing, and the concentration of alcohol used had no effect on the crazing stress value.

The sensitivity of crazing to stress in PMMA after machining was influenced by the alcohol concentration. As the alcohol concentration increased from 75% to 95%, the stress sensitivity of oriented PMMA nonlinearly increased from 0.02 to 0.035 mm/MPa and then to 0.055 mm/MPa. The higher the alcohol concentration in oriented PMMA, the more sensitive crazing was to stress, resulting in the faster development of cracks. In the regular PMMA, the sensitivity of crazing to stress increased from 0.065 to 0.077 mm/MPa after treatment with 75% alcohol. The increase in sensitivity was observed as the tensile stress increased to 28.5 MPa, indicating that the presence of alcohol enhanced the development of crazing in PMMA.

Both alcohol solvents and machine damage can promote crazing initiation and progression. The physical diffusion of the alcohol solvent lowers the crazing stress threshold, whereas machining raises the stress of PMMA by creating concentrated stress points. When stress is superimposed on the surface of PMMA, the crazing of PMMA becomes extremely sensitive to tensile stress. The forward extension of the crazing tip and the thickening of the crazing simultaneously occur in the crazing growth process of oriented PMMA, and a dynamic balance can be achieved throughout the crazing growth process. When the two factors are applied to the surface of regular PMMA, the stress gradient limits the thickening of crazing. The forward extension of the crazing tip is continuously strengthened as the tensile stress increases. Additionally, the direction of the crazing tip growth is uncontrollable due to the different positions of the concentrated stress generated by the two factors. The network structure of an oriented PMMA molecular chain is more uniform after directional stretching, which effectively reduces the stress gradient that is generated by the concentrated stress and causes crazing to grow in a fixed direction. 

## 4. Conclusions

The tensile experiments were used to analyze the crazing processes of oriented and regular PMMA after exposure to solvent atmospheres and machining. We determined that the solvent atmosphere could affect the initiation and growth of crazing in PMMA through the concentration and stress gradients generated by physical molecular diffusion. Machining also contributed to the crazing process by creating localized areas of concentrated stress. Both treatments had the potential to substantially decrease the anti-crazing stress in PMMA and enhance its sensitivity to stress during the growth of crazing. Maintaining uniform stress and strain is an effective means of reducing the crazing caused by machining and solvent environments. We also delved into the molecular chain change process and the behavior of PMMA under various influences during the crazing process and ultimately concluded the following:

- Physical diffusion causes swelling points on the surface of PMMA due to the effect of the solvent atmosphere. Regular PMMA surface swelling points are distributed in 1–3 μm spots, whereas oriented PMMA surface swelling points are distributed in 20–40 μm strips. At the swelling points, concentration and stress gradients arise; this increases the growth rate of the crazing tip and reduces the stress on PMMA against crazing.
- Machining primarily affects the growth of crazing through residual stress. The application of tensile stress leads to localized plastic deformation around areas of concentrated stress, resulting in the formation of micro-cavities. These micro-cavities eventually merge to form crazing.
- The tip of the crazing in regular PMMA severely bends due to the uneven distribution of molecular chains. Orientated PMMA, however, can effectively alleviate the residual stress that is caused by machining through directional stretching. The direction of tensile stress is always perpendicular to the direction of crazing growth.
- The critical stress threshold for PMMA crazing is diminished by both machining and exposure to solvent atmospheres. On average, the initiation stress of crazing was reduced by 20%, and the sensitivity of crazing to stress was elevated by 0.35–0.45 mm/MPa.

## Figures and Tables

**Table 1:** Partial material properties of regular and oriented PMMA.

Property	Regular PMMA	Oriented PMMA
Tensile Strength at 23 °C (MPa)	77.8	94.6
Tensile Strength at 100 °C (MPa)	1.84	28.4
Bending strength (MPa)	130	>205
Impact strength (kJm^–2^)	22.6	57.0
Break elongation (%)	4.0	7.7

**Table 2:** Experimental parameters.

Group No.	Material	Machining	Solvent	Load
Group 1	regular PMMA	Non-milling	95% alcohol by volume	Standing
Group 2	oriented PMMA	Non-milling	95% alcohol by volume	Standing
Group 3	regular PMMA	Non-milling	Air	Uniaxial tension
Group 4	oriented PMMA	Non-milling	95% alcohol by volume	Uniaxial tension
Group 5	regular PMMA	Milling	95% alcohol by volume	Standing
Group 6	oriented PMMA	Milling	95% alcohol by volume	Standing
Group 7	regular PMMA	Milling	Air	Uniaxial tension
Group 8	regular PMMA	Milling	75% alcohol by volume	Uniaxial tension
Group 9	regular PMMA	Milling	95% alcohol by volume	Uniaxial tension
Group 10	oriented PMMA	Milling	Air	Uniaxial tension
Group 11	oriented PMMA	Milling	75% alcohol by volume	Uniaxial tension
Group 12	oriented PMMA	Milling	95% alcohol by volume	Uniaxial tension

**Table 3:** Crazing growth state of PMMA with 95% alcohol.

Materials and Treatment	Stress at Crazing	Sensitivity of Crazing to Stress
Regular PMMA	31.4 MPa	0.02 mm/MPa
Regular PMMA with 95% alcohol	29.8 MPa	0.049 mm/MPa
Oriented PMMA	51.4 MPa	0.015 mm/MPa
Oriented PMMA with 95% alcohol	43.4 MPa	0.055 mm/MPa

**Table 4:** Crazing growth state of PMMA before and after machining.

Materials and Treatment	Stress at Crazing	Sensitivity of Crazing to Stress
Regular PMMA	31.4 MPa	0.02 mm/MPa
Regular PMMA after milling	26.8 MPa	0.065 mm/MPa
Oriented PMMA	51.4 MPa	0.015 mm/MPa
Oriented PMMA after milling	42.2 MPa	0.04 mm/MPa

**Table 5:** The crazing growth state of PMMA with the combined action of alcohol and machining.

Material and Treatment	Stress at Crazing	Sensitivity of Crazing to Stress
Oriented PMMA	42.2 MPa	0.02 mm/MPa
Oriented PMMA with 75% alcohol	31.8 MPa	0.035 mm/MPa
Oriented PMMA with 95% alcohol	31.4 MPa	0.055 mm/MPa
Regular PMMA	26.8 MPa	0.065 mm/MPa
Regular PMMA with 75% alcohol	23.4 MPa	0.077 mm/MPa
Regular PMMA with 95% alcohol	22.8 MPa	0.12 mm/MPa

## Data Availability

Data sharing is not applicable to this article.
